# Prevalence and correlates of anxiety and depression in caregivers to assisted living residents during COVID-19: a cross-sectional study

**DOI:** 10.1186/s12877-022-03294-y

**Published:** 2022-08-12

**Authors:** Natasha E. Lane, Matthias Hoben, Joseph E. Amuah, David B. Hogan, Jennifer Baumbusch, Andrea Gruneir, Stephanie A. Chamberlain, Lauren E. Griffith, Kimberlyn M. McGrail, Kyle Corbett, Colleen J. Maxwell

**Affiliations:** 1 ICES, Toronto, ON Canada; 2 Department of Internal Medicine, University of British Columbia, Vancouver, BC Canada; 3 Faculty of Nursing, College of Health Sciences, University of Alberta, Edmonton, AB Canada; 4 School of Epidemiology and Public Health, University of Ottawa, Ottawa, ON Canada; 5 Division of Geriatric Medicine, Department of Medicine, Cumming School of Medicine, University of Calgary, Calgary, AB Canada; 6 School of Nursing, University of British Columbia, Vancouver, BC Canada; 7 Department of Family Medicine, Faculty of Medicine and Dentistry, College of Health Sciences, University of Alberta, Edmonton, AB Canada; 8 Department of Health Research Methods, Evidence and Impact, Faculty of Health Sciences, McMaster University, Hamilton, ON Canada; 9 School of Population and Public Health, Centre for Health Services and Policy Research, University of British Columbia, Vancouver, BC Canada; 10 School of Pharmacy, University of Waterloo, ON N2L 3G1 Waterloo, Canada; 11 School of Public Health Sciences, University of Waterloo, Waterloo, ON Canada

**Keywords:** Assisted living, Caregivers, COVID-19, Mental health

## Abstract

**Background:**

Family and friend caregivers play significant roles in advocating for and ensuring quality health and social care of residents in Assisted Living (AL) homes. However, little is known about how the COVID-19 pandemic and related visitor restrictions affected their health and mental well-being. We examined the prevalence and correlates of anxiety and depressive symptoms among caregivers of AL residents during the initial wave of COVID-19 in two Canadian provinces.

**Methods:**

A cross-sectional web-based survey was conducted among family/friend caregivers of AL residents in Alberta and British Columbia (Oct 28, 2020—Mar 31, 2021) to collect data on their sociodemographic, health and caregiving characteristics, as well as concerns about residents’ health and social care before and during the first wave of the pandemic. A clinically significant anxiety disorder and depressive symptoms were assessed with the GAD-7 and CES-D10 instruments, respectively. Separate multivariable (modified) Poisson regression models identified caregiver correlates of each mental health condition.

**Results:**

Among the 673 caregivers completing the survey (81% for Alberta residents), most were women (77%), white (90%) and aged ≥ 55 years (81%). Clinically significant anxiety and depression were present in 28.6% and 38.8% of caregivers respectively. Both personal stressors (comorbidity level, income reduction, low social support) and caregiving stressors exacerbated by the pandemic were independently associated with caregiver anxiety and depression. The latter included increased concern about the care recipients’ depression (adjusted risk ratio [adjRR] = 1.84, 95% confidence interval [CI] 1.19–2.85 for caregiver anxiety and adjRR = 1.75, 95% CI 1.26–2.44 for caregiver depressive symptoms) and reported intention to withdraw the resident from AL because of COVID-19 (adjRR = 1.24, 95%CI 0.95–1.63 for caregiver anxiety and adjRR = 1.37, 95%CI 1.13–1.67 for caregiver depressive symptoms).

**Conclusions:**

Caregivers of residents in AL homes reported significant personal and caregiving-related stressors during the initial wave of COVID-19 that were independently associated with an increased likelihood of experiencing clinically significant anxiety and depressive symptoms. Healthcare providers and AL staff should be aware of the prevalence and varied correlates of caregivers’ mental health during public health crises so that appropriate screening and support may identified and implemented.

**Supplementary Information:**

The online version contains supplementary material available at 10.1186/s12877-022-03294-y.

## Background

Family and friend caregivers (hereafter referred to as caregivers) provide significant unpaid emotional, physical, and practical support to persons with age-related needs [1, 2]. Though they experience many benefits from caregiving, they are also at increased risk for poorer physical and psychological well-being compared with non-caregiving peers [1–4]. The COVID-19 pandemic significantly heightened the potential for caregiver distress, anxiety and/or mood disorders [2, 5]. This may be especially so for caregivers of older residents in nursing homes and assisted living (AL), given the overwhelming burden of COVID-19 cases and deaths [6] and the impact of strict visitor restrictions in these settings [5, 7].

Relative to nursing homes [6, 8–10], research on the impact of COVID-19 on the health and well-being of AL residents and their caregivers is scarce [11, 12]. A cross-sectional survey of 84 caregivers of Alberta AL residents showed a five-fold increase (13.4% to 67.1%) in self-reported moderate-severe anxiety (assessed with the State Anxiety Scale) during the first wave of COVID-19 [5]. Though direct comparisons are problematic, these estimates are higher than the self-reported changes in high anxiety (5% to 20%) and depression (4% to 10%) noted in a cross-sectional survey of the general Canadian population [13].

A more rigorous examination of the epidemiology of anxiety and mood disorders among AL resident caregivers during the COVID-19 pandemic is warranted. AL homes represent approximately 40 percent of residential care beds [11, 14] and their numbers are growing across Canada [15] and the United States [11, 16]. AL residents are of advanced age (average 84 years) and exhibit high rates of dementia (≥ 60%) [17, 18], other mental health conditions (34% with depression) and multimorbidity [18, 19]. However, relative to nursing homes, AL settings offer fewer services and have fewer skilled staff members per resident and no onsite 24-h registered nurses. As such, they are more dependent on caregiver involvement than nursing homes. Research has demonstrated the significant contributions of caregivers to the health, social and psychological well-being of AL residents [11, 18, 20], and visitor restrictions during COVID-19 undoubtedly had deleterious effects on the mental well-being of both residents and their caregivers [21, 22]. Importantly, caregivers experiencing anxiety and/or depression have higher health care needs and are less able to continue caregiving [23].

This study aimed to identify the prevalence and correlates of clinically significant anxiety and depressive symptoms among caregivers of AL residents during the first wave of COVID-19 in two Canadian provinces. Guided by relevant conceptual models [24, 25], we hypothesized that after adjusting for predisposing characteristics, the experience of pandemic-related stressors, including income loss, as well as stressors relevant to caregiving (e.g., increased concern about resident’s mental well-being) would independently increase caregivers’ risk of both mental health conditions.

## Methods

This study was conducted in accordance with the principles of the Declaration of Helsinki and received ethics approval by the Health Research Ethics Board at the University of Alberta (Pro00101048), University of Calgary Conjoint Health Research Ethics Board (REB20-1544), Human Research Ethics Board at the University of British Columbia (H20-01732) and University of Waterloo Human Research Ethics Committee (ORE#42494). Operational approvals from participating AL homes were obtained.

It is reported per STROBE guidelines (Table S1, Additional File 1) [26].

### Study design and setting

This cross-sectional study is part of a prospective cohort investigation, COVCARES-AB/BC *(COVID-19 and Caregivers of Assisted living Residents: their Experiences and Support needs*), underway in Alberta and British Columbia, Canada**. **Both provinces initiated “essential visitors” policies in AL in mid-March 2020, allowing a single caregiver to enter for end-of-life visits or to provide assistance with feeding or mobility [27]. However, the decision to impose more stringent or complete bans on visitors during the first wave was at the discretion of individual homes.

### Study sample

We invited all eligible AL homes in Alberta and British Columbia to participate in COVCARES-AB/BC. Eligibility criteria included homes that were licensed and publicly subsidized (in Alberta these are known as designated supportive living), in operation for at least 6 months, not primarily serving psychiatric clients and with a minimum number of residents aged 65 + years (4 for small, 10 for large homes, based on region’s median bed-size). Based on available provincial registries (listing all publicly subsidized AL homes in the two provinces), we identified 163 eligible homes in Alberta and 137 in British Columbia at study onset. We contacted each eligible AL home by email and invited a key contact (e.g., facility administrator or director of care) to participate in our study. We sent up to three reminder emails in two-week intervals and contacted remaining non-respondents by telephone. Further description about AL in the two provinces is provided in Table S2, Additional File 1.

Consenting homes distributed our study materials and open web-based survey link to all identified caregivers via their internal email listing and/or in-person for caregivers visiting the home. This survey link was also distributed via social media, websites, email lists and newsletters affiliated with our study team and government/caregiver stakeholder partners in both provinces. Those who identified as the primary adult caregiver (i.e., aged 18 + years and most informed or involved in care) of a resident aged 65 + years who had lived in the AL home for three or more months prior to March 1, 2020, were eligible to participate.

The baseline caregiver survey was administered online, between October 28, 2020, and March 31, 2021, by the Survey Research Centre (SRC) at the University of Waterloo. The SRC has been in operation for over 20 years and employs robust and standardized methods, training, and protocols for survey research. These robust measures ensured that fraudulent survey completions were minimized (and tracked for removal) and that any duplicate and ineligible survey responses were identified and removed from the data. Caregivers completing the survey received a $25 coffee gift card.

### Measures

Our caregiver survey included standardized and validated measures used in an earlier study of AL and nursing home residents and their caregivers in Alberta [18, 28] and by the Canadian Longitudinal Study on Aging [29, 30]. Included were items regarding caregivers’ sociodemographic characteristics, physical and mental health status, and social support. Items capturing changes in the frequency and nature of their visits with residents and involvement in resident care (during the 3-months prior to and post March 1/20) were also included. We selected this timeframe as it encompasses the first wave of the pandemic, during which visitor restrictions were the most stringent. Survey measures of pandemic-related stressors included loss of employment and income (and concern level), absence of opportunities to stay well-informed/engaged in care of the resident, and increased levels of concern about resident’s physical and mental well-being.

#### Anxiety and depressive symptoms

Caregiver anxiety was assessed with the 7-item Generalized Anxiety Disorder scale (GAD-7) [31, 32]. The items capture how often (over past 2 weeks) respondents have been bothered by feelings associated with anxiety. We used a cut-point of ≥ 8 to define a clinically significant anxiety disorder (sensitivity 92%, specificity 70%) [33].

Depressive symptoms were assessed with the Center for Epidemiologic Studies Depression Scale, Short Form (CES-D10) [34] that includes 10 items regarding how often (in past week) respondents have experienced signs or symptoms of depression. We used a cut-point of ≥ 10 to identify clinically significant depressive symptoms (sensitivity 89%, specificity 47% for major depressive disorder) [35].

Both instruments have been extensively used in previous caregiver studies [29, 30, 36, 37]. Consistent with scale development [31, 32, 34], respondents with missing values for only one item were assigned the mean of their other item responses before totalling their scale score. The terms ‘anxiety’ and ‘depressive symptoms’ are used in the remaining text to reflect significant symptomatology.

#### Covariates

Based on the Stress Process Model [24], background factors examined included caregiver age, gender, marital status, ethnicity, highest education, pre-pandemic household income, and province of AL home. Exacerbating and ameliorating factors [25] examined included caregivers’ number of chronic conditions, and perceived emotional/informational social support. The latter was assessed using the relevant subscale of the RAND Medical Outcomes Study – Social Support Survey [MOS-SSS]) [38].

We also examined pandemic-specific stressors supported by the Appraisal Model [25], including change in employment status and income reduction (combined with level of concern) during the 3 months post-March 1, 2020. Covariates reflecting caregiving stressors included their belief as to whether the AL home/staff created opportunities for them to be well-informed and involved in care of the resident, the change (comparing the 3 months post- vs pre-March 1/20) in their level of concern about the resident’s depression status, and whether they considered moving the resident out of the AL home during the 3 months post-March 1/20 because of COVID-19 [39].

AL home characteristics, including bed size, urban/rural location and ownership status (private for-profit vs non-profit) were also examined.

### Statistical analysis

Descriptive analyses examined the distribution of caregivers’ characteristics, overall and by the presence/absence of anxiety or depressive symptoms.

We employed unadjusted and adjusted Poisson regression models, modified for binary outcomes [40], to examine associations between caregivers’ characteristics and our two mental health outcomes.

Guided by our theoretical models [24, 25], we developed our final models in a staged approach by first including caregivers’ background and health characteristics, followed by general and caregiving-specific pandemic related stressors and lastly emotional/informational social support. Each covariate was added one at a time while examining for potential collinearity issues. To provide final parsimonious multivariable models, we removed statistically non-significant factors, but retained those significant for only one outcome (but not the other) for comparative purposes. For both outcomes, we retained household income (though non-significant in full models) to allow for an interpretation of pandemic-related income loss in the context of baseline socioeconomic status. We did not include self-rated health in our models, given its conceptual overlap with our two mental health outcome measures. Though our survey asked whether caregivers tested positive for COVID-19 during the first pandemic wave, it was not feasible to explore this measure in our models as only three caregivers responded positively to this question.

#### Missing data analysis

For most covariates, the proportion of respondents with missing values was small (e.g., < 1–2%). For household income, the proportion was larger (13.7%), so we allowed missing values to represent one level of this measure. Missing values were more common for anxiety and depressive symptoms (e.g., 75 and 54 respondents with 2 + missing scale items for the GAD-7 and CES-D10, respectively), so we compared the distribution of caregiver characteristics by the presence/absence of missing data for these two outcomes. As missing values were also relatively more common for emotional/informational social support (*n* = 41), we showed final models with and without this measure.

#### Sensitivity analyses

We assessed whether accounting for clustering of caregivers within AL homes affected model estimates by including a robust sandwich variance estimator in our models [41]. Intra-cluster correlations were small (0.009 for anxiety, 0.000 for depression) and *p *values of the home-level covariance component were not statistically significant, suggesting no meaningful home-level clustering. We also conducted multiple imputation analyses using the fully conditional method and specifying a generalized logit distribution [42, 43].

All analyses were two-tailed with statistical significance defined as *p* ≤ *0.05*. SAS version 9.4 (SAS Institute Inc., Cary, NC) was used to conduct all analyses.

## Results

Among the 673 caregivers (associated with 134 AL homes) who completed the survey, 546 (81.1%) cared for AL residents in Alberta. For eight caregivers, the name and location of the AL home was not provided. About two thirds of caregivers were affiliated with urban or large AL homes and they were approximately equally distributed by home ownership status (54.6% for profit; 45.4% non-profit). Given our recruitment strategy and open survey link, it is not possible to accurately identify the total number of caregivers who were made aware of the survey link, thus it is not possible to calculate contact, cooperation or response rates. We estimate our study sample represented approximately 7% of all potentially eligible primary caregivers of AL residents in the 134 AL homes (e.g., there were about 11,017 beds in the 134 AL homes at the time of our study, assuming an occupancy rate of 95%, a rate of 4% of residents with no family member/friend and that each of the remaining residents had one primary caregiver meeting our eligibility criteria, 673/10,047 = 7%).

Respondents were primarily women, married, white and aged ≥ 55 years (Table 1). As expected, most (62%) were daughters. Though about half of respondents indicated having one or more chronic conditions, most (89%) reported being in good to excellent health. Respondents generally reported having high emotional/information social support (78%). Just over a quarter reported reduced income because of COVID-19 and among this group, 83% (149/179) indicated they were concerned about this income loss (Table 2). Approximately 24% of caregivers indicated that they did not believe the AL home or staff created opportunities for them to be well-informed and/or involved in the care of the resident during the early months of the pandemic and 21% considered moving the resident out of the AL home during this time (only 17/139 did so). Over a third reported an increase in their level of concern about the resident’s depression status (from being not, slightly or somewhat concerned in the 3 months pre-March 01, 2020, to being moderately or extremely concerned in the subsequent 3 months).

**Table 1:** Distribution of sociodemographic and health characteristics, overall and by presence of clinically significant anxiety disorder and depressive symptoms among AL caregivers

Characteristic	Overall (*N* = 673) Column % (n)	Clinically Significant Anxiety Disorder		Depressive Symptoms	
		**Present (28.6%; 171/598)** **Col % (n)**	**Absent (71.4%; 427/598)** **Col % (n)**	**Present (38.8%; 240/619)** **Col % (n)**	**Absent (61.2%; 379/619)** **Col % (n)**
Province (Location of Home)					
Alberta	81.1 (546)	81.9 (140)	80.3 (343)	81.3 (195)	81.0 (307)
British Columbia	18.9 (127)	18.1 (31)	19.7 (84)	18.8 (45)	19.0 (72)
Age					
18–44	6.4 (43)	12.3 (21) ^‡^	3.8 (16)	10.0 (24) ^*^	4.8 (18)
45–54	12.2 (82)	11.7 (20)	12.9 (55)	11.7 (28)	12.7 (48)
55–64	42.3 (284)	44.4 (76)	42.5 (181)	43.8 (105)	41.5 (157)
65 +	39.1 (262)	31.6 (54)	40.9 (174)	34.6 (83)	41.0 (155)
Gender					
Woman	76.8 (515)	83.6 (143) ^*^	73.9 (315)	82.9 (199) ^†^	72.5 (274)
Man / Prefer Not to Answer	23.3 (156)	16.4 (28)	26.1 (111)	17.1 (41)	27.5 (104)
Marital Status					
Married / Common-law	83.1 (555)	85.3 (145)	83.7 (355)	81.2 (194)	85.1 (320)
Other	16.9 (113)	14.7 (25)	16.3 (69)	18.8 (45)	14.9 (56)
Relationship to Resident					
Spouse / Parent ^a^	5.8 (39)	7.6 (13)^*^	5.2 (22)	7.5 (18)^†^	4.5 (17)
Daughter (including in-law)	62.0 (417)	69.6 (119)	58.8 (251)	67.5 (162)	58.3 (221)
Son (including in-law)	16.5 (111)	10.5 (18)	18.3 (78)	10.0 (24)	19.8 (75)
Sibling	7.3 (49)	4.7 (8)	8.7 (37)	6.3 (15)	8.4 (32)
Friend / Neighbour /Other	8.5 (57)	7.6 (13)	9.2 (39)	8.8 (21)	9.0 (34)
Ethnicity					
White	89.9 (598)	91.7 (155)	90.5 (383)	90.8 (216)	90.9 (341)
Non-White	10.1 (67)	8.3 (14)	9.5 (40)	9.2 (22)	9.1 (34)
Highest Education					
University	31.0 (205)	25.4 (43)^*^	36.2 (153)	30.4 (72)	34.1 (128)
College / Trade	42.5 (281)	43.2 (73)	40.4 (171)	43.0 (102)	41.6 (156)
High School or Less	26.6 (176)	31.4 (53)	23.4 (99)	26.6 (63)	24.3 (91)
Household Income (before Mar 1/20)					
> $100,000	27.3 (184)	28.1 (48)	29.5 (126)	26.7 (64)	29.8 (113)
$80—$99,000	15.0 (101)	12.3 (21)	17.1 (73)	13.3 (32)	16.1 (61)
$50-$79,000	23.8 (160)	22.8 (39)	23.0 (98)	22.5 (54)	25.6 (97)
< $50,000	20.2 (136)	23.4 (40)	17.1 (73)	25.0 (60)	16.6 (63)
missing	13.7 (92)	13.5 (23)	13.4 (57)	12.5 (30)	11.9 (45)
# Chronic Conditions					
None	43.0 (288)	39.4 (67) ^†^	45.9 (196)	39.5 (94) ^‡^	44.9 (170)
1–2	41.6 (279)	38.2 (65)	44.0 (188)	38.7 (92)	45.7 (173)
3 +	11.6 (78)	18.8 (32)	8.4 (36)	17.7 (42)	7.1 (27)
Don’t know / Prefer not to answer	3.7 (25)	3.5 (6)	1.6 (7)	4.2 (10)	2.4 (9)
Self-rated Health					
Excellent	16.9 (113)	7.1 (12) ^‡^	21.4 (91)	8.0 (19) ^‡^	23.3 (88)
Very Good	40.5 (271)	28.2 (48)	46.0 (196)	30.1 (72)	47.9 (181)
Good	31.8 (213)	42.4 (72)	25.8 (110)	40.6 (97)	24.1 (91)
Fair / Poor	10.9 (73)	22.4 (38)	6.8 (29)	21.3 (51)	4.8 (18)
Emotional / Informational Social Support					
High	78.0 (493)	62.4 (101) ^‡^	84.3 (349)	63.6 (147) ^‡^	86.5 (314)
Low	22.0 (139)	37.6 (61)	15.7 (65)	36.4 (84)	13.5 (49)

^*^ *p* ≤ 0.05

^†^ *p* ≤ 0.01

^‡^ *p* ≤ 0.001

^a^ 95% were spouse of resident

**Table 2:** Distribution of COVID-19 pandemic-related stressors, overall and by presence of clinically significant anxiety disorder and depressive symptoms among AL caregivers

Stressor	Overall (*N* = 673) Column % (n)	Clinically Significant Anxiety Disorder		Depressive Symptoms	
		**Present (28.6%; 171/598)** **Col % (n)**	**Absent (71.4%; 427/598)** **Col % (n)**	**Present (38.8%; 240/619)** **Col % (n)**	**Absent (61.2%; 379/619)** **Col % (n)**
Income Reduction (3 months post Mar 1/20) & Level of Concern					
No	73.4 (494)	69.0 (118)^‡^	77.1 (329)	65.0 (156)^‡^	77.6 (294)
Yes, Not concerned	4.5 (30)	3.5 (6)	4.2 (18)	3.8 (9)	4.8 (18)
Yes, Somewhat concerned	14.1 (95)	9.9 (17)	15.9 (68)	14.6 (35)	14.8 (56)
Yes, Very/Extremely concerned	8.0 (54)	17.5 (30)	2.8 (12)	16.7 (40)	2.9 (11)
Change in Employment Status (3 months post Mar 1/20)					
No	84.0 (562)	77.1 (131)^†^	87.3 (371)	78.2 (187)^*^	86.2 (325)
Yes	16.0 (107)	22.9 (39)	12.7 (54)	21.8 (52)	13.8 (52)
Believe home/staff created opportunities to be well-informed / involved in care of resident					
Yes	75.6 (506)	67.7 (115)^†^	78.8 (335)	68.2 (163)^‡^	80.6 (304)
No	24.4 (163)	32.4 (55)	21.2 (90)	31.8 (76)	19.4 (73)
Considered moving resident out of home (3 months post Mar 1/20)					
No	79.3 (532)	69.8 (118)^‡^	82.2 (351)	68.9 (164)^‡^	84.7 (321)
Yes	20.7 (139)	30.2 (51)	17.8 (76)	31.1 (74)	15.3 (58)
Change in caregiver’s concern about resident’s depression (3 months post vs pre-Mar 1/20)					
Remained not concerned/slightly-somewhat concerned	31.6 (210)	17.3 (29)^‡^	36.3 (154)	18.6 (44)^‡^	38.3 (144)
Increased to slightly/somewhat concerned	18.5 (123)	18.5 (31)	18.9 (80)	15.3 (36)	20.7 (78)
Remained moderately/extremely concerned	12.9 (86)	13.1 (22)	12.7 (54)	15.7 (37)	11.4 (43)
Increased to moderately concerned	15.0 (100)	18.5 (31)	14.6 (62)	18.6 (44)	13.3 (50)
Increased to extremely concerned	22.0 (146)	32.7 (55)	17.5 (74)	31.8 (75)	16.2 (61)

^*^ *p* ≤ 0.05

^†^ *p* ≤ 0.01

^‡^ *p* ≤ 0.001

Anxiety was present in 28.6% of caregivers and 38.8% exhibited depressive symptoms. There was considerable overlap in these outcomes (e.g., 85.6% of caregivers with anxiety also had depressive symptoms vs. 18.6% among those without anxiety). Both anxiety and depressive symptoms were significantly more common in caregivers who were younger (aged 18–44), women, a spouse or daughter of the resident, and those who reported being very or extremely concerned about pandemic-related income loss, having 3 or more chronic conditions, and receiving low emotional/informational social support (Table 3). Caregivers who reported experiencing pandemic-related caregiving stresses (i.e., not being kept well-informed and/or involved in the care of the resident, considered moving the resident out of the AL home, and/or increased concern about resident’s depression levels) were also significantly more likely to exhibit both mental health outcomes. Caregivers with lower education were significantly more likely to have anxiety (but not depressive symptoms), whereas those reporting low household income were significantly more likely to have depressive symptoms.

**Table 3:** Unadjusted risk ratios (95% confidence interval) for clinically significant anxiety disorder and depressive symptoms associated with AL caregiver characteristics

Characteristic	Clinically Significant Anxiety Disorder		Depressive Symptoms	
	**% with Disorder**	**Unadj RR (95% CI)**	**% with Symptoms**	**Unadj RR (95% CI)**
**Total Sample**	**28.6**		**38.8**	
Age				
18–44	56.8	**2.40 (1.66–3.45)**	57.1	**1.64 (1.20–2.24)**
45–54	26.7	1.13 (0.72–1.75)	36.8	1.06 (0.75–1.49)
55–64	29.6	1.25 (0.93–1.69)	40.1	1.15 (0.91–1.44)
65 + (ref group)	23.7	1.00	34.9	1.00
Gender				
Woman	31.2	**1.55 (1.08–2.22)**	42.1	**1.49 (1.12–1.97)**
Man / Prefer Not to Answer (ref group)	20.1	1.00	28.3	1.00
Relationship to Resident				
Spouse / Parent	37.1	**1.98 (1.09–3.61)**	51.4	**2.12 (1.32–3.41)**
Daughter (including in-law)	32.2	**1.72 (1.10–2.67)**	42.3	**1.74 (1.21–2.52)**
Son (including in-law) (ref group)	18.8	1.00	24.2	1.00
Sibling	17.8	0.95 (0.45–2.01)	31.9	1.32 (0.76–2.27)
Friend / Neighbour / Other	25.0	1.33 (0.71–2.50)	38.2	1.58 (0.97–2.56)
Highest Education				
University (ref group)	21.9	1.00	36.0	1.00
College / Trade	29.9	1.36 (0.98–1.89)	39.5	1.10 (0.87–1.39)
High School or Less	34.9	**1.59 (1.13–2.24)**	40.9	1.14 (0.87–1.48)
Household Income (before Mar 1/20)				
> $100,000 (ref group)	27.6	1.00	36.2	1.00
$80—$99,000	22.3	0.81 (0.52–1.27)	34.4	0.95 (0.68–1.34)
$50-$79,000	28.5	1.03 (0.72–1.48)	35.8	0.99 (0.74–1.32)
< $50,000	35.4	1.28 (0.91–1.81)	48.8	**1.35 (1.03–1.76)**
missing	28.8	1.04 (0.68–1.59)	40.0	1.11 (0.79–1.55)
Income Reduction (3 months post Mar 1/20) & Level of Concern				
No (ref group)	26.4	1.00	34.7	1.00
Yes, Not concerned	25.0	0.95 (0.47–1.93)	33.3	0.96 (0.56–1.66)
Yes, Somewhat concerned	20.0	0.76 (0.48–1.19)	38.5	1.11 (0.83–1.48)
Yes, Very/Extremely concerned	71.4	**2.71 (2.12–3.46)**	78.4	**2.26 (1.87–2.74)**
Change in Employment Status (3 months post Mar 1/20)				
No (ref group)	26.1	1.00	36.5	1.00
Yes	41.9	**1.61 (1.21–2.13)**	50.0	**1.37 (1.09–1.71)**
# Chronic Conditions				
None (ref group)	25.5	1.00	35.6	1.00
1–2	25.7	1.01 (0.75–1.35)	34.7	0.98 (0.77–1.23)
3 +	47.1	**1.85 (1.33–2.56)**	60.9	**1.71 (1.33–2.19)**
Don’t know / Prefer not to answer	46.2	1.81 (0.97–3.38)	52.6	1.48 (0.94–2.33)
Believe home/staff created opportunities to be well-informed / involved in care of resident				
Yes (ref group)	25.6	1.00	34.9	1.00
No	37.9	**1.48 (1.14–1.93)**	51.0	**1.46 (1.20–1.79)**
Considered moving resident out of home (3 months post Mar 1/20)				
No (ref group)	25.2	1.00	33.8	1.00
Yes	40.2	**1.60 (1.23–2.08)**	56.1	**1.66 (1.36–2.02)**
Change in caregiver’s concern about resident’s depression (3 months post vs pre-Mar 1/20)				
Remained not concerned/slightly-somewhat concerned (ref group)	15.9	1.00	23.4	1.00
Increased to slightly/somewhat concerned	27.9	**1.76 (1.13–2.76)**	31.6	1.35 (0.93–1.96)
Remained moderately/extremely concerned	29.0	**1.83 (1.12–2.97)**	46.3	**1.98 (1.39–2.81)**
Increased to moderately concerned	33.3	**2.10 (1.35–3.27)**	46.8	**2.00 (1.43–2.80)**
Increased to extremely concerned	42.6	**2.69 (1.82–3.97)**	55.2	**2.36 (1.75–3.18)**
Emotional / Informational Social Support				
High (ref group)	22.4	1.00	31.9	
Low	48.4	**2.16 (1.68–2.77)**	63.2	**1.98 (1.64–2.39)**

With some exceptions, the above caregiver characteristics remained significantly associated with both mental health outcomes in our final multivariable models (Table 4). Caregiver age (being younger, Model B: adjRR = 1.93, 95% CI 1.21–3.09 for caregivers aged 18–44 vs 65 + years) and gender (being a woman, adjRR = 1.32, 95% CI 1.01–1.73) showed a significant association with anxiety and depressive symptoms, respectively. Caregivers with lower education showed an increased risk for anxiety (adjRR = 1.62, 95% CI 1.14–2.29 for ≤ high school vs. university level) and those who considered moving the resident out of the AL home showed an increased risk for depressive symptoms (adjRR = 1.37, 95% CI 1.13–1.67). Significant associations with both mental health outcomes were observed for caregivers who reported being very or extremely concerned about pandemic-related income loss (adjRR = 1.72, 95% CI 1.23–2.42 for anxiety, adjRR = 1.69, 95% CI 1.32–2.17 for depressive symptoms), 3 or more chronic conditions (adjRR = 1.87, 95% CI 1.31–2.66 for anxiety, adjRR = 1.56, 95% CI 1.17–2.06 for depressive symptoms), becoming more concerned about the resident’s depression (e.g., adjRR = 1.84, 95% CI 1.19–2.85 for anxiety, adjRR = 1.75, 95% CI 1.26–2.44 for depressive symptoms comparing those who became extremely concerned vs. remained not or only slightly concerned) and low emotional/information social support (adjRR = 1.87, 95% CI 1.44–2.44 for anxiety, adjRR = 1.67, 95% CI 1.38–2.04 for depressive symptoms). Several variables (relationship to the resident, reported change in employment and absence of opportunities to remain well-informed/involved in resident care) were not significantly associated with either outcome after covariate adjustment, reflecting their strong correlations with other covariates. None of the AL home characteristics examined were significantly associated with either anxiety or depressive symptoms.

**Table 4:** Adjusted risk ratios (95% confidence interval) for clinically significant anxiety disorder and depressive symptoms associated with AL caregiver characteristics

Characteristic	Clinically Significant Anxiety Disorder Adj RR (95% CI)		Depressive Symptoms Adj RR (95% CI)	
	**Model A **^a^	**Model B **^b^	**Model A **^c^	**Model B **^d^
Age				
18–44	**1.80 (1.15–2.83)**	**1.93 (1.21–3.09)**	1.18 (0.83–1.70)	1.20 (0.82–1.74)
45–54	1.14 (0.73–1.79)	1.28 (0.80–2.03)	0.92 (0.64–1.33)	0.97 (0.67–1.42)
55–64	1.10 (0.82–1.49)	1.18 (0.85–1.62)	1.02 (0.81–1.27)	1.03 (0.82–1.30)
65 + (ref group)				
Gender				
Woman	1.29 (0.91–1.84)	1.25 (0.87–1.79)	1.29 (0.99–1.69)	**1.32 (1.01–1.73)**
Man / Prefer Not to Answer (ref group)				
Highest Education				
University (ref group)				
College / Trade	**1.37 (1.01–1.85)**	**1.36 (1.00–1.84)**	1.05 (0.83–1.31)	1.09 (0.87–1.37)
High School or Less	**1.65 (1.17–2.32)**	**1.62 (1.14–2.29)**	1.14 (0.87–1.49)	1.19 (0.91–1.55)
Household Income (before Mar 1/20)				
> $100,000 (ref group)				
$80—$99,000	0.79 (0.51–1.22)	0.74 (0.48–1.14)	0.92 (0.66–1.28)	0.88 (0.64–1.22)
$50-$79,000	1.00 (0.70–1.42)	0.99 (0.69–1.42)	0.99 (0.74–1.33)	0.93 (0.69–1.26)
< $50,000	1.02 (0.71–1.48)	0.92 (0.62–1.36)	1.19 (0.90–1.59)	1.17 (0.88–1.57)
missing	1.07 (0.69–1.66)	1.06 (0.67–1.68)	1.18 (0.83–1.66)	1.21 (0.86–1.70)
Income Reduction (3 months post Mar 1/20) & Level of Concern				
No (ref group)				
Yes, Not concerned	1.00 (0.52–1.91)	1.16 (0.61–2.22)	1.15 (0.72–1.85)	1.31 (0.82–2.09)
Yes, Somewhat concerned	0.80 (0.51–1.25)	0.82 (0.53–1.27)	1.19 (0.89–1.58)	1.19 (0.90–1.58)
Yes, Very/Extremely concerned	**1.93 (1.42–2.63)**	**1.72 (1.23–2.42)**	**1.91 (1.52–2.41)**	**1.69 (1.32–2.17)**
# Chronic Conditions				
None (ref group)				
1–2	1.08 (0.81–1.45)	1.06 (0.79–1.43)	1.01 (0.81–1.27)	0.99 (0.79–1.24)
3 +	**1.93 (1.37–2.72)**	**1.87 (1.31–2.66)**	**1.61 (1.23–2.12)**	**1.56 (1.17–2.06)**
Don’t know / Prefer not to answer	1.44 (0.75–2.74)	1.19 (0.63–2.25)	1.16 (0.73–1.86)	1.05 (0.68–1.63)
Considered moving resident out of home (3 months post Mar 1/20)				
No (ref group)	1.25 (0.95–1.65)	1.24 (0.95–1.63)	**1.39 (1.14–1.70)**	**1.37 (1.13–1.67)**
Yes				
Change in caregiver’s concern about resident’s depression (3 months post vs pre-Mar 1/20)				
Remained not concerned/slightly-somewhat concerned (ref group)				
Increased to slightly/somewhat concerned	**1.61 (1.03–2.52)**	1.42 (0.90–2.25)	1.21 (0.84–1.75)	1.19 (0.82–1.72)
Remained moderately/extremely concerned	1.53 (0.94–2.49)	1.43 (0.87–2.36)	**1.63 (1.15–2.32)**	**1.68 (1.17–2.40)**
Increased to moderately concerned	**1.91 (1.24–2.96)**	**1.66 (1.06–2.59)**	**1.72 (1.23–2.41)**	**1.65 (1.17–2.32)**
Increased to extremely concerned	**2.07 (1.36–3.15)**	**1.84 (1.19–2.85)**	**1.85 (1.34–2.53)**	**1.75 (1.26–2.44)**
Emotional / Informational Social Support				
High (ref group)		**1.87 (1.44–2.44)**		**1.67 (1.38–2.04)**
Low				

^a^ Estimates from multivariable (modified) Poisson regression model adjusting for all variables in column, total missing from model A = 89

^b^ Estimates from multivariable (modified) Poisson regression model adjusting for all variables in column, total missing from model B = 111

^c^ Estimates from multivariable (modified) Poisson regression model adjusting for all variables in column, total missing from model A = 71

^d^ Estimates from multivariable (modified) Poisson regression model adjusting for all variables in column, total missing from model B = 95

As model findings remained robust to both sensitivity analyses, we have reported final models not adjusting for clustering or incorporating our multiple imputation analyses (see Tables S3 & S4, Additional file 1 for missing data analyses and multiple imputation model estimates).

## Discussion

Insufficient research and policy attention has been directed to the impact of COVID-19 on the health and well-being of caregivers of older adults residing in AL homes. We showed that among caregivers of AL residents in two Canadian provinces, over a quarter had anxiety and over a third, depressive symptoms. In keeping with our a priori hypothesis, we found that anxiety and depressive symptoms were associated with a combination of caregiver characteristics and pandemic-related personal and caregiving stressors. After adjusting for predisposing factors, caregivers experiencing a concerning loss of income and/or increased concern about the resident’s depression during the early period of the pandemic were significantly more likely to have anxiety and depressive symptoms. Those who reported that they considered moving their family member/friend out of AL during this period were also more likely to exhibit both mental health conditions, though the association was more pronounced for depressive symptoms.

While the prevalence of anxiety and depression increased during the pandemic among both caregivers and non-caregivers [13] baseline rates were higher among the former [3]. In the Canadian Longitudinal Study on Aging (CLSA), the prevalence of CES-D10 defined depression among middle-age and older adults increased from 16.4% at baseline (2011–2015) to 22.0% (fall 2020) [30]. Our higher prevalence estimates parallel other self-reported data on depression and anxiety among caregivers of persons in residential care during the pandemic [22]. Though direct comparisons are challenging due to heterogeneity in study measures, settings and population characteristics, it appears that the pandemic was associated with worse mental health outcomes among caregivers in particular [5, 29, 44].

In our study, younger caregivers and those with lower education were significantly more likely to experience anxiety, while women were more likely to exhibit depressive symptoms. These findings align with extant evidence on the relevance of age, gender and lower socioeconomic status to worse mental health during the pandemic among the general population [30, 45], at-risk sub-groups (e.g., college students) [46], and caregivers specifically [29, 47]. Other studies have also found that those with multiple chronic conditions [48] and relatively low emotional/informational social support [47, 49] were significantly more like to have both anxiety and depression during the COVID-19 pandemic.

In addition, we found that pandemic-specific personal and caregiving stressors were associated with an increased likelihood of caregiver anxiety and depression. As in our study, Raina and colleagues showed that a reported loss of income during the pandemic was independently associated with worse mental health among CLSA participants [30]. Earlier data from a small AL sample demonstrated that caregivers of AL residents expressed worry that pandemic-related isolation and loss of contact with familiar persons could hasten declines in the psychological and cognitive health of residents [5, 50]. More than a third of the caregivers we surveyed reported increased concern about their care recipient becoming more depressed during the first COVID-19 wave and those who did were significantly more likely to have anxiety and depressive symptoms. Caregivers who reported they considered moving the resident out of the AL home early in the pandemic were also more likely to have depression and to a lesser extent, anxiety. Though less than three percent actually followed through with this, our findings likely reflect caregivers’ increased concern and stress about the resident’s health worsening should they remain in the home as well as the adequacy of available resources to deal with resident’s care needs [25].

Strengths of this study include its use of comprehensive primary data with validated measures of anxiety and depressive symptoms, and its large sample size spanning two Canadian provinces. It addresses a key knowledge gap as AL caregivers typically comprise a minority of samples studied to date [5, 51], or were excluded altogether [44, 49, 52].

There are limitations to consider. As this is a cross-sectional study we are unable to comment on the temporal nature of observed associations, though our finding are consistent with previous longitudinal research [4, 30]. Under various assumptions, we estimate that our study sample represented approximately 7% of all potentially eligible primary caregivers of AL residents among included AL homes (and a relatively lower proportion of all potentially eligible primary AL caregivers in both provinces) and thus our findings may not be fully generalizable to this larger target population. The participants were mostly white, English-speaking women with disproportionately high socioeconomic status. The exclusion of caregivers from diverse cultures and the likelihood that some who declined participation did so because of various stressors, limits the generalizability of our results and may have resulted in an underestimation of anxiety and/or depressive symptoms. We did not have data on care recipient characteristics (e.g., whether tested positive for COVID-19) that may be relevant to caregiver mental health. Finally, the cut-points we used for anxiety and depressive symptoms prioritized sensitivity over specificity, increasing the likelihood for false positives.

## Conclusions

The COVID-19 pandemic exposed weaknesses in how we care for older adults in congregate care settings. Inadequate attention has been paid to how these care issues and pandemic-related stressors affected their caregivers. Our findings demonstrate that while caregivers of AL residents were subject to the same stressors about their own health and income as the general population, they carried the extra burden of worrying about the health of their family member or friend in AL. Healthcare providers and AL staff should be aware of the prevalence and correlates of mental health issues affecting caregivers during public health and other crises. An enhanced understanding of these issues could inform effective approaches to screening for mental health conditions and permit the implementation of targeted support to caregivers adversely affected. One promising approach that could be easily modified to ensure support and mental health services for caregivers during crises is the Residential Care Transition Module (RCTM) [53]. This psychosocial intervention, originally developed by researchers in the United States, incorporates telehealth as a means for supporting caregivers during transition of their family member to residential care [53]. Though further research is warranted, preliminary findings [54] suggest that similar types of interventions have potential to mitigate adverse mental health consequences of public health emergencies on caregivers of AL residents.

## Supplementary Information

**Additional file 1: Table S1.** STROBE guidelines for reporting observational (cross-sectional) studies. **Table S2.** Descriptionof AL in Alberta and British Columbia, Canada. **Table S3.** Distribution of AL caregiver characteristics, overall and by missing responses for clinically significant anxiety disorder and depressive symptoms. **Table S4.** Adjusted risk ratios (95% confidence interval) for clinically significant anxiety disorder and depressive symptoms associated with AL caregiver characteristics [following Multiple Imputation of Missing Data]. 

## Data Availability

All study data and analytical output are held on a secure server at the University of Alberta as per relevant Ethics Committees’ guidelines. As such, all study datasets are not publicly available. Access is restricted to approved study investigators and research associates. Aggregate data and relevant statistical code are available from the primary investigators on reasonable request. For additional information regarding the availability of data contact Dr. Colleen Maxwell, email: colleen.maxwell@uwaterloo.ca.

## References

[1] Arriagada P. The experiences and needs of older caregivers in Canada. Ottawa, ON: Statistics Canada 2020. https://publications.gc.ca/collections/collection_2020/statcan/75-006-x/75-006-2020-7-eng.pdf. Accessed 22 Mar 2022.

[2] The Ontario Caregiver Association. 3rd Annual Spotlight on Ontario’s Caregivers: COVID-19 Edition, 2020. https://ontariocaregiver.ca/publications/oco-spotlight-report/. Accessed 22 Mar 2022.

[3] Pinquart M, Sörensen S (2003). Differences between caregivers and noncaregivers in psychological health and physical health: a meta-analysis. Psychol Aging.

[4] Del-Pino-Casado R, Priego-Cubero E, López-Martínez C, Orgeta V (2021). Subjective caregiver burden and anxiety in informal caregivers: A systematic review and meta-analysis. PLoS ONE.

[5] Anderson S, Parmar J, Dobbs B, Tian PGJ (2021). A Tale of Two Solitudes: Loneliness and Anxiety of Family Caregivers Caring in Community Homes and Congregate Care. Int J Environ Res Public Health.

[6] Comas-Herrera A, Zalakain J, Litwin C, Hsu A, Lane N, Fernández J. Mortality associated with COVID-19 outbreaks in care homes: early international evidence. Article in LTCcovid.org, International Long-Term Care Policy Network, CPEC-LSE, 21 May 2020. https://ltccovid.org/wp-content/uploads/2020/06/Mortality-associated-with-COVID-21-May-3.pdf. Accessed 22 Mar 2022.

[7] Gaugler JE, Mitchell LL (2022). Reimagining Family Involvement in Residential Long-Term Care. J Am Med Dir Assoc.

[8] Verbeek H, Gerritsen DL, Backhaus R, de Boer BS, Koopmans R, Hamers JPH (2020). Allowing Visitors Back in the Nursing Home During the COVID-19 Crisis: A Dutch National Study Into First Experiences and Impact on Well-Being. J Am Med Dir Assoc.

[9] Wammes JD, Kolk D, van den Besselaar JH, MacNeil-Vroomen JL, Buurman-van Es BM, van Rijn M (2020). Evaluating Perspectives of Relatives of Nursing Home Residents on the Nursing Home Visiting Restrictions During the COVID-19 Crisis: A Dutch Cross-Sectional Survey Study. J Am Med Dir Assoc.

[10] Hado E, Friss FL (2020). Amid the COVID-19 Pandemic, Meaningful Communication between Family Caregivers and Residents of Long-Term Care Facilities is Imperative. J Aging Soc Policy.

[11] Zimmerman S, Sloane PD, Katz PR, Kunze M, O'Neil K, Resnick B (2020). The Need to Include Assisted Living in Responding to the COVID-19 Pandemic. J Am Med Dir Assoc.

[12] Coe NB, Van Houtven CH (2020). Living Arrangements of Older Adults and COVID-19 Risk: It Is Not Just Nursing Homes. J Am Geriatr Soc.

[13] Dozois  DJA (2021). Mental Health Research Canada. Anxiety and depression in Canada during the COVID-19 pandemic: A national survey. Can Psychol.

[14] Harris-Kojetin L, Sengupta M, Park-Lee E, Valverde R, Caffrey C, Rome V, et al. Long-Term Care Providers and services users in the United States: data from the National Study of Long-Term Care Providers, 2013–2014. Vital Health Stat 3*.* 2016; (38): x-xii; 1–105. 27023287

[15] Alberta Health Services. Alberta Health Services Annual Report 2018–19: Alberta Health Services 2020. https://www.albertahealthservices.ca/assets/about/publications/2018-19-annual-report-web-version.pdf. Accessed 22 Mar 2022.

[16] Vipperman A, Zimmerman S, Sloane PD (2021). COVID-19 Recommendations for Assisted Living: Implications for the Future. J Am Med Dir Assoc.

[17] Zimmerman S, Sloane PD, Reed D (2014). Dementia prevalence and care in assisted living. Health Aff.

[18] Strain LA, Maxwell CJ, Wanless D, Gilbart E. Designated Assisted Living (DAL) and Long-Term Care (LTC) in Alberta: Selected Highlights from the Alberta Continuing Care Epidemiological Studies (ACCESS). Edmonton, AB: ACCES Research Group, University of Alberta, 2011. http://hdl.handle.net/10402/era.23779. Accessed 30 Mar 2022.

[19] Hogan DB, Amuah JE, Strain LA, Wodchis WP, Soo A, Eliasziw M (2014). High rates of hospital admission among older residents in assisted living facilities: opportunities for intervention and impact on acute care. Open Med.

[20] Port CL, Zimmerman S, Williams CS, Dobbs D, Preisser JS, Williams SW. Families filling the gap: comparing family involvement for assisted living and nursing home residents with dementia. Gerontologist. 2005; 45 Spec No 1(1): 87–95. 10.1093/geront/45.suppl_1.87 16230755

[21] Kemp CL (2021). #MoreThanAVisitor: Families as "Essential" Care Partners During COVID-19. Gerontologist.

[22] Nash  WA, Harris  LM, Heller  KE, Mitchell  BD (2021). "We Are Saving Their Bodies and Destroying Their Souls.": Family Caregivers' Experiences of Formal Care Setting Visitation Restrictions during the COVID-19 Pandemic. J Aging Soc Policy.

[23] Fekete C, Tough H, Siegrist J, Brinkhof MW (2017). Health impact of objective burden, subjective burden and positive aspects of caregiving: an observational study among caregivers in Switzerland. BMJ Open.

[24] Pearlin LI, Mullan JT, Semple SJ, Skaff MM (1990). Caregiving and the stress process: an overview of concepts and their measures. Gerontologist.

[25] Sörensen S, Conwell Y (2011). Issues in dementia caregiving: effects on mental and physical health, intervention strategies, and research needs. Am J Geriatr Psychiatry.

[26] von Elm E, Altman DG, Egger M, Pocock SJ, Gøtzsche PC, Vandenbroucke JP (2014). The Strengthening the Reporting of Observational Studies in Epidemiology (STROBE) Statement: guidelines for reporting observational studies. Int J Surg.

[27] North American Observatory on Health Systems and Policies. North American COVID-19 Policy Response Monitor: Alberta, 2020. https://ihpme.utoronto.ca/wp-content/uploads/2020/08/AB-COVID19-Response-Monitor_20200807.pdf. Accessed 30 Mar 2022.

[28] Wanless D, Strain LA, Maxwell CJ. Designated Assisted Living (DAL) and Long-Term Care (LTC) in Alberta: Alberta Continuing Care Epidemiological Studies (ACCES) Methodology. Edmonton, AB: ACCES Research Group, University of Alberta, 2011. http://hdl.handle.net/10402/era.23788. Accessed 30 Mar 2022.

[29] Wister A, Li L, Mitchell B, Wolfson C, McMillan J, Griffith LE (2022). Levels of depression and anxiety among informal caregivers during the COVID-19 pandemic: A study based on the Canadian Longitudinal Study on Aging. J Gerontol B Psychol Sci Soc Sci.

[30] Raina P, Wolfson C, Griffith L, Kirkland S, McMillan J, Basta N (2021). A longitudinal analysis of the impact of the COVID-19 pandemic on the mental health of middle-aged and older adults from the Canadian Longitudinal Study on Aging. Nature Aging.

[31] Spitzer RL, Kroenke K, Williams JB, Löwe B (2006). A Brief Measure for Assessing Generalized Anxiety Disorder: The GAD-7. Arch Intern Med.

[32] Kroenke K, Spitzer RL, Williams JB, Monahan PO, Löwe B (2007). Anxiety disorders in primary care: prevalence, impairment, comorbidity, and detection. Ann Intern Med.

[33] Johnson SU, Ulvenes PG, Øktedalen T, Hoffart A (2019). Psychometric Properties of the General Anxiety Disorder 7-Item (GAD-7) Scale in a Heterogeneous Psychiatric Sample. Front Psychol.

[34] Andresen EM, Malmgren JA, Carter WB, Patrick DL (1994). Screening for depression in well older adults: evaluation of a short form of the CES-D (Center for Epidemiologic Studies Depression Scale). Am J Prev Med.

[35] Björgvinsson T, Kertz SJ, Bigda-Peyton JS, McCoy KL, Aderka IM (2013). Psychometric Properties of the CES-D-10 in a Psychiatric Sample. Assessment.

[36] Andresen EM, Byers K, Friary J, Kosloski K, Montgomery R (2013). Performance of the 10-item Center for Epidemiologic Studies Depression scale for caregiving research. SAGE Open Med.

[37] Li Q, Zhang H, Zhang M, Li T, Ma W, An C (2021). Mental Health Multimorbidity among Caregivers of Older Adults During the COVID-19 Epidemic. Am J Geriatr Psychiatry.

[38] Sherbourne CD, Stewart AL (1991). The MOS social support survey. Soc Sci Med.

[39] Picard A. If you can get your relatives out of seniors’ homes, try to do so as fast as you can. The Globe and Mail 2020 April 2, 2020. https://www.theglobeandmail.com/canada/article-if-you-can-get-your-relatives-out-of-seniors-homes-try-to-do-so-as/. Accessed 30 Mar 2022.

[40] Zou G (2004). A modified poisson regression approach to prospective studies with binary data. Am J Epidemiol.

[41] Zou GY, Donner A (2013). Extension of the modified Poisson regression model to prospective studies with correlated binary data. Stat Methods Med Res.

[42] van Buuren S (2007). Multiple imputation of discrete and continuous data by fully conditional specification. Stat Methods Med Res.

[43] Rubin DB. Multiple imputation for nonresponse in surveys: John Wiley & Sons; 1987.

[44] Beach SR, Schulz R, Donovan H, Rosland AM (2021). Family Caregiving During the COVID-19 Pandemic. Gerontologist.

[45] Statistics Canada. Canadians' mental health during the COVID-19 pandemic Ottawa, ON: Statistics Canada 2020. https://www150.statcan.gc.ca/n1/en/daily-quotidien/200527/dq200527b-eng.pdf?st=LSbxwkDJ. Accessed 30 Mar 2022.

[46] Appleby JA, King N, Saunders KE, Bast A, Rivera D, Byun J (2022). Impact of the COVID-19 pandemic on the experience and mental health of university students studying in Canada and the UK: a cross-sectional study. BMJ Open.

[47] Allen J, Uekusa S, Alpass FM (2022). Longitudinal cohort study of depression and anxiety among older informal caregivers following the initial COVID-19 pandemic response in Aotearoa New Zealand. J Aging Health.

[48] Wu T, Jia X, Shi H, Niu J, Yin X, Xie J (2021). Prevalence of mental health problems during the COVID-19 pandemic: A systematic review and meta-analysis. J Affect Disord.

[49] Messina A, Lattanzi M, Albanese E, Fiordelli M (2022). Caregivers of people with dementia and mental health during COVID-19: findings from a cross-sectional study. BMC Geriatr.

[50] Parmar J, Anderson S, Dobbs B, Tian PGJ, Charles L, Triscott J (2021). Neglected Needs of Family Caregivers during the COVID-19 Pandemic and What They Need Now: A Qualitative Study. Diseases.

[51] Lee Y, Bierman A, Penning M (2020). Psychological Well-Being Among Informal Caregivers in the Canadian Longitudinal Study on Aging: Why the Location of Care Matters. J Gerontol B Psychol Sci Soc Sci.

[52] Zucca M, Isella V, Lorenzo RD, Marra C, Cagnin A, Cupidi C (2021). Being the Family Caregiver of a Patient With Dementia During the Coronavirus Disease 2019 Lockdown. Front Aging Neurosci.

[53] Gaugler JE, Statz TL, Birkeland RW, Louwagie KW, Peterson CM, Zmora R (2020). The Residential Care Transition Module: a single-blinded randomized controlled evaluation of a telehealth support intervention for family caregivers of persons with dementia living in residential long-term care. BMC Ger.

[54] Brooks D, Beattie E, Edwards H, Fielding E, Gaugler JE (2021). Pilot study of the Residential Care Transition Module to support Australian spouses of people with dementia. Clin Geron.

