# Supplementary material for: Prevalence and correlates of anxiety and depression in caregivers to assisted living residents during COVID-19: a cross-sectional study
Source: BMC Geriatr. 2022 Aug 12;22:662. doi: 10.1186/s12877-022-03294-y (PMC9372518; doi:10.1186/s12877-022-03294-y)
Supplement: Supplementary file 1 — Additional file 1: Table S1. STROBE guidelines for reporting observational (cross-sectional) studies. Table S2. Descriptionof AL in Alberta and British Columbia, Canada. Table S3. Distribution of AL caregiver characteristics, overall and by missing responses for clinically significant anxiety disorder and depressive symptoms. Table S4. Adjusted risk ratios (95% confidence interval) for clinically significant anxiety disorder and depressive symptoms associated with AL caregiver characteristics [following Multiple Imputation of Missing Data]. [file 12877_2022_3294_MOESM1_ESM.docx]

**Additional File 1**

**Prevalence and Correlates of Anxiety and Depression in Caregivers to Assisted Living Residents During COVID-19: A Cross-sectional Study**

**Lane et al. June 23, 2022**

**Table S1.** STROBE guidelines for reporting observational (cross-sectional) studies.

**Table S2.** Description of AL in Alberta and British Columbia, Canada.

**Table S3**. Distribution of AL caregiver characteristics, overall and by missing responses for clinically significant anxiety disorder and depressive symptoms.

**Table S4.** Adjusted risk ratios (95% confidence interval) for clinically significant anxiety disorder and depressive symptoms associated with AL caregiver characteristics [following Multiple Imputation of Missing Data].

**Table S1.** STROBE guidelines for reporting observational (cross-sectional) studies.

|  | Item No | Recommendation | Page No |
| --- | --- | --- | --- |
| **Title and abstract** | 1 | (*a*) Indicate the study’s design with a commonly used term in the title or the abstract | 1,2 |
|  |  | (*b*) Provide in the abstract an informative and balanced summary of what was done and what was found | 2,3 |
| Introduction | | | |
| Background/rationale | 2 | Explain the scientific background and rationale for the investigation being reported | 4,5 |
| Objectives | 3 | State specific objectives, including any prespecified hypotheses | 5 |
| Methods | | | |
| Study design | 4 | Present key elements of study design early in the paper | 5 |
| Setting | 5 | Describe the setting, locations, and relevant dates, including periods of recruitment, exposure, follow-up, and data collection | 6 |
| Participants | 6 | (*a*) Give the eligibility criteria, and the sources and methods of selection of participants | 6 |
| Variables | 7 | Clearly define all outcomes, exposures, predictors, potential confounders, and effect modifiers. Give diagnostic criteria, if applicable | 7-8 |
| Data sources/ measurement | 8* | For each variable of interest, give sources of data and details of methods of assessment (measurement). Describe comparability of assessment methods if there is more than one group | 7,8 |
| Bias | 9 | Describe any efforts to address potential sources of bias | 9 |
| Study size | 10 | Explain how the study size was arrived at | 6 |
| Quantitative variables | 11 | Explain how quantitative variables were handled in the analyses. If applicable, describe which groupings were chosen and why | 7,8 |
| Statistical methods | 12 | (*a*) Describe all statistical methods, including those used to control for confounding | 8,9 |
|  |  | (*b*) Describe any methods used to examine subgroups and interactions | N/A |
|  |  | (*c*) Explain how missing data were addressed | 9 |
|  |  | (*d*) If applicable, describe analytical methods taking account of sampling strategy | N/A |
|  |  | (*e*) Describe any sensitivity analyses | 9,10 |
| Results | | | |
| Participants | 13* | (a) Report numbers of individuals at each stage of study—eg numbers potentially eligible, examined for eligibility, confirmed eligible, included in the study, completing follow-up, and analysed | 10 |
|  |  | (b) Give reasons for non-participation at each stage | N/A |
|  |  | (c) Consider use of a flow diagram |  |
| Descriptive data | 14* | (a) Give characteristics of study participants (eg demographic, clinical, social) and information on exposures and potential confounders | 10,11 Tables 1,2 |
|  |  | (b) Indicate number of participants with missing data for each variable of interest | 9, Table S3 |
| Outcome data | 15* | Report numbers of outcome events or summary measures | 11 |
| Main results | 16 | (*a*) Give unadjusted estimates and, if applicable, confounder-adjusted estimates and their precision (eg, 95% confidence interval). Make clear which confounders were adjusted for and why they were included | 11,12  Tables 3,4 |
|  |  | (*b*) Report category boundaries when continuous variables were categorized |  |
|  |  | (*c*) If relevant, consider translating estimates of relative risk into absolute risk for a meaningful time period |  |
| Other analyses | 17 | Report other analyses done—eg analyses of subgroups and interactions, and sensitivity analyses | 12 |
| Discussion | | | |
| Key results | 18 | Summarise key results with reference to study objectives | 12 |
| Limitations | 19 | Discuss limitations of the study, taking into account sources of potential bias or imprecision. Discuss both direction and magnitude of any potential bias | 14 |
| Interpretation | 20 | Give a cautious overall interpretation of results considering objectives, limitations, multiplicity of analyses, results from similar studies, and other relevant evidence | 13-15 |
| Generalisability | 21 | Discuss the generalisability (external validity) of the study results | 14 |
| Other information | | | |
| Funding | 22 | Give the source of funding and the role of the funders for the present study and, if applicable, for the original study on which the present article is based | Title page |

*Give information separately for exposed and unexposed groups.

**Table S2.** **Description of AL in Alberta and British Columbia, Canada.**

| **Alberta** | **British Columbia [BC]** |
| --- | --- |
| - In Alberta, there are publicly subsidized and private pay AL operators. - Publicly funded (subsidized) AL in Alberta is known as designated supportive living (SL). - Under the Supportive Living Accommodation Licensing Act, SL must be licensed for resident safety and security and also comply with Accommodation Standards; designated SL must further comply with the Continuing Care Health Service Standards. - Designated SL is composed of 3 progressive levels of support moving from the lowest level of support available (SL3) to higher levels of support (SL4 and SL4-Dementia)   - SL3 is for individuals who are medically & physically stable and can move independently or move with limited assistance. Health care aides are available on site 24hrs/day and other health care services are scheduled and provided by home care.   - SL4 is for individuals with more complex health needs and who might require assistance with eating & transfers. It has health care aides and a licensed practical nurse available 24hrs/day, and other care needs (e.g., rehabilitation therapy) are contracted through home care.   - SL 4D is like SL4 but is specifically for individuals with moderate to severe dementia. - Further details about Designated SL in Alberta can be found here:   <https://www.albertahealthservices.ca/cc/Page15490.aspx>  <https://www.albertahealthservices.ca/cc/page15328.aspx> | - In BC, there are publicly subsidized and private pay AL operators. - Under the Community Care and Assisted Living Act (which sets minimum health and safety requirements) – all publicly subsidized and private pay AL operators that meet the definition of an AL residence under the Act are required to be registered with the provincial AL registrar. - Publicly subsidized AL services provide housing, hospitality and one or two personal assistance services (prescribed services) for adults who can live independently and make decisions on their own behalf but require a supportive environment due to physical and functional health challenges.   - Eligibility require resident to be able to make decisions on their own behalf or who has a spouse willing to live with the resident and is willing/able to make decisions on their behalf.   - If the resident requires three or more personal assistance services they are not eligible for publicly subsidized AL. - BC does not differentiate various levels of AL depending on individuals’ care needs. - Further details about AL in BC can be found here:   <https://www2.gov.bc.ca/gov/content/health/accessing-health-care/home-community-care/care-options-and-cost/assisted-living>  <https://www2.gov.bc.ca/gov/content/health/accessing-health-care/finding-assisted-living-or-residential-care/residential-care-facilities/finding-a-residential-care-facility> |

**Table S3. Distribution of AL caregiver characteristics, overall and by missing responses for clinically significant anxiety disorder and depressive symptoms.**

| **Characteristic** | **Overall**  **(N=673)**  **Column % (n)** | **Clinically Significant Anxiety Disorder** | | **Depressive Symptoms** | |
| --- | --- | --- | --- | --- | --- |
|  |  | **Not missing**  **(88.9%; 598/673)**  **Col % (n)** | **Missing**  **(11.1%; 75/673)**  **Col % (n)** | **Not missing**  **(92.0%; 619/673)**  **Col % (n)** | **Missing**  **(8.0%; 54/673)**  **Col % (n)** |
| Province (Location of Home)  Alberta  British Columbia | 81.1 (546)  18.9 (127) | 80.8 (483)  19.2 (115) | 84.0 (63)  16.0 (12) | 81.1 (502)  18.9 (117) | 81.5 (44)  18.5 (10) |
| Age  18-44  45-54  55-64  65+ | 6.4 (43)  12.2 (82)  42.3 (284)  39.1 (262) | 6.2 (37)  12.6 (75)  43.1 (257)  38.2 (228) | 8.1 (6)  9.5 (7)  36.5 (27)  46.0 (34) | 6.8 (42)  12.3 (76)  42.4 (262)  38.5 (238) | 1.9 (1)  11.3 (6)  41.5 (22)  45.3 (24) |
| Gender  Woman  Man / Prefer Not to Answer | 76.8 (515)  23.3 (156) | 76.7 (458)  23.3 (139) | 77.0 (57)  23.0 (17) | 76.5 (473)  23.5 (145) | 79.3 (42)  20.8 (11) |
| Marital Status  Married / Common-law  Other | 83.1 (555)  16.9 (113) | 84.2 (500)^*^  15.8 (94) | 74.3 (55)  25.7 (19) | 83.6 (514)  16.4 (101) | 77.4 (41)  22.6 (12) |
| Relationship to Resident  Spouse / Parent  Daughter (including in-law)  Son (including in-law)  Sibling  Friend / Neighbour  Other | 5.8 (39)  62.0 (417)  16.5 (111)  7.3 (49)  3.4 (23)  5.1 (34) | 5.9 (35)  61.9 (370)  16.1 (96)  7.5 (45)  3.2 (19) 5.5 (33) | 5.3 (4)  62.7 (47)  20.0 (15)  5.3 (4)  5.3 (4)  1.3 (1) | 5.7 (35)  61.9 (383)  16.0 (99)  7.6 (47)  3.7 (23) 5.2 (32) | 7.4 (4)  63.0 (34)  22.2 (12)  3.7 (2)  0.0 (0)  3.7 (2) |
| Ethnicity  White  Non-White | 89.9 (598)  10.1 (67) | 90.9 (538)^*^  9.1 (54) | 82.2 (60)  17.8 (13) | 90.9 (557)^†^  9.1 (56) | 78.9 (41)  21.2 (11) |
| Highest Education  University  College / Trade  High School or Less | 31.0 (205)  42.5 (281)  26.6 (176) | 33.1 (196)^†^  41.2 (244)  25.7 (152) | 12.9 (9)  52.9 (37)  34.3 (24) | 32.7 (200)^‡^  42.2 (258)  25.2 (154) | 10.0 (5)  46.0 (23)  44.0 (22) |
| Household Income (before Mar 1/20)  >$100,000  $80 - $99,000  $50-$79,000  <$50,000  missing | 27.3 (184)  15.0 (101)  23.8 (160)  20.2 (136)  13.7 (92) | 29.1 (174)^†^  15.7 (94)  22.9 (137)  18.9 (113)  13.4 (80) | 13.3 (10)  9.3 (7)  30.7 (23)  30.7 (23)  16.0 (12) | 28.6 (177)^‡^  15.0 (93)  24.4 (151)  19.9 (123)  12.1 (75) | 13.0 (7)  14.8 (8)  16.7 (9)  24.1 (13)  31.5 (17) |
| Income Reduction (3 months post Mar 1/20) & Level of Concern  No  Yes, Not concerned  Yes, Somewhat concerned  Yes, Very/Extremely concerned | 73.4 (494)  4.5 (30)  14.1 (95)  8.0 (54) | 74.8 (447)^*^  4.0 (24)  14.2 (85)  7.0 (42) | 62.7 (47)  8.0 (6)  13.3 (10)  16.0 (12) | 72.7 (450)  4.4 (27)  14.7 (91)  8.2 (51) | 81.5 (44)  5.6 (3)  7.4 (4)  5.6 (3) |
| Change in Employment Status (3 months post Mar 1/20)  No  Yes | 84.0 (562)  16.0 (107) | 84.4 (502)  15.6 (93) | 81.1 (60)  18.9 (14) | 83.1 (512)^*^  16.9 (104) | 94.3 (50)  5.7 (3) |
| # Chronic Conditions  None  1-2  3+  DK / Prefer not to answer | 43.0 (288)  41.6 (279)  11.6 (78)  3.7 (25) | 44.1 (263)^‡^  42.4 (253)  11.4 (68)  2.9 (13) | 34.3 (25)  35.6 (26)  13.7 (10)  16.4 (12) | 42.8 (264)^†^  43.0 (265)  11.2 (69)  3.1 (19) | 45.3 (24)  26.4 (14)  17.0 (9)  11.3 (6) |
| Self-rated Health  Excellent  Very Good  Good  Fair / Poor | 16.9 (113)  40.5 (271)  31.8 (213)  10.9 (73) | 17.3 (103)  40.9 (244)  30.5 (182)  11.2 (67) | 13.5 (10)  36.5 (27)  41.9 (31)  8.1 (6) | 17.3 (107)  41.0 (253)  30.5 (188)  11.2 (69) | 11.3 (6)  34.0 (18)  47.2 (25)  7.6 (4) |
| Emotional / Informational Social Support  High  Low | 78.0 (493)  22.0 (139) | 78.1 (450)  21.9 (126) | 76.8 (43)  23.2 (13) | 77.6 (461)  22.4 (133) | 84.2 (32)  15.8 (6) |
|  |  |  |  |  |  |
| Believe home/staff created opportunities to be well-informed / involved in care of resident  Yes  No | 75.6 (506)  24.4 (163) | 75.6 (450)  24.4 (145) | 75.7 (56)  24.3 (18) | 75.8 (467)  24.2 (149) | 73.6 (39)  26.4 (14) |
| Considered moving resident out of home (3 months post Mar 1/20)  No  Yes | 79.3 (532)  20.7 (139) | 78.7 (469)  21.3 (127) | 84.0 (63)  16.0 (12) | 78.6 (485)  21.4 (132) | 87.0 (47)  13.0 (7) |
| Change in caregiver’s concern about resident’s depression (3 months post vs pre-Mar 1/20)  Remained not concerned/slightly- somewhat concerned  Increased to slightly/somewhat concerned  Remained moderately/extremely concerned  Increased to moderately concerned  Increased to extremely concerned | 31.6 (210)  18.5 (123)  12.9 (86)  15.0 (100)  22.0 (146) | 30.9 (183)  18.8 (111)  12.8 (76)  15.7 (93)  21.8 (129) | 37.0 (27)  16.4 (12)  13.7 (10)  9.6 (7)  23.3 (17) | 30.7 (188)  18.6 (114)  13.1 (80)  15.4 (94)  22.2 (136) | 41.5 (22)  17.0 (9)  11.3 (6)  11.3 (6)  18.9 (10) |
|  |  |  |  |  |  |

*p≤0.05; †p≤0.01; ‡p≤0.001

**Table S4. Adjusted risk ratios (95% confidence interval) for clinically significant anxiety disorder and depressive symptoms associated with AL caregiver characteristics [following Multiple Imputation of Missing Data].**

| **Characteristic** | **Clinically Significant Anxiety Disorder**  **Adj RR (95% CI)** | | **Depressive Symptoms**  **Adj RR (95% CI)** | |
| --- | --- | --- | --- | --- |
|  | **Model A** | **Model B** | **Model A** | **Model B** |
| Age  18-44  45-54  55-64  65+ (ref group) | **1.90 (1.10-2.69)**  1.16 (0.67-1.64)  1.20 (0.85-1.55) | **1.90 (1.08-2.72)**  1.23 (0.71-1.74)  1.18 (0.83-1.53) | 1.18 (0.77-1.60)  0.95 (0.62-1.29)  1.04 (0.81-1.27) | 1.20 (0.77-1.62)  1.01 (0.66-1.36)  1.04 (0.81-1.26) |
| Gender  Woman  Man / Prefer Not to Answer (ref group) | 1.28 (0.85-1.71) | 1.34 (0.88-1.79) | 1.29 (0.95-1.64) | 1.35 (0.99-1.70) |
| Highest Education  University (ref group)  College / Trade  High School or Less | 1.36 (0.96-1.75)  **1.65 (1.09-2.20)** | 1.39 (0.99-1.78)  **1.69 (1.12-2.27)** | 1.07 (0.83-1.31)  1.12 (0.82-1.42) | 1.09 (0.85-1.34)  1.16 (0.85-1.46) |
| Household Income (before Mar 1/20)  >$100,000 (ref group)  $80 - $99,000  $50-$79,000  <$50,000  missing | 0.78 (0.45-1.11)  1.00 (0.65-1.34)  0.98 (0.64-1.32)  1.02 (0.63-1.42) | 0.72 (0.42-1.02)  0.95 (0.62-1.28)  0.91 (0.59-1.23)  1.04 (0.64-1.45) | 0.92 (0.63-1.22)  0.96 (0.68-1.24)  1.15 (0.83-1.47)  1.10 (0.75-1.46) | 0.87 (0.60-1.15)  0.92 (0.66-1.19)  1.08 (0.78-1.38)  1.11 (0.75-1.46) |
| Income Reduction (3 months post Mar 1/20) & Level of Concern  No (ref group)  Yes, Not concerned  Yes, Somewhat concerned  Yes, Very/Extremely concerned | 0.94 (0.30-1.58)  0.88 (0.51-1.25)  **2.01 (1.45-2.57)** | 1.03 (0.34-1.72)  0.87 (0.52-1.23)  **1.78 (1.26-2.31)** | 1.08 (0.56-1.60)  1.13 (0.81-1.45)  **1.83 (1.42-2.23)** | 1.18 (0.62-1.73)  1.12 (0.81-1.43)  **1.65 (1.26-2.03)** |
| # Chronic Conditions  None (ref group)  1-2  3+  DK / Prefer not to answer | 1.08 (0.78-1.38)  **1.95 (1.30-2.61)**  1.40 (0.48-2.32) | 1.04 (0.75-1.32)  **1.88 (1.25-2.51)**  1.21 (0.42-2.00) | 1.01 (0.78-1.23)  **1.66 (1.22-2.10)**  1.25 (0.70-1.80) | 0.97 (0.76-1.19)  **1.61 (1.18-2.05)**  1.12 (0.64-1.60) |
| Considered moving resident out of home (3 months post Mar 1/20)  No (ref group)  Yes | 1.21 (0.89-1.53) | 1.15 (0.85-1.45) | **1.37 (1.09-1.64)** | **1.31 (1.06-1.57)** |
| Change in caregiver’s concern about resident’s depression (3 months post vs pre-Mar 1/20)  Remained not concerned/slightly- somewhat concerned (ref group)  Increased to slightly/somewhat concerned  Remained moderately/extremely concerned  Increased to moderately concerned  Increased to extremely concerned | 1.53 (0.87-2.20)  1.45 (0.78-2.12)  **1.74 (1.02-2.45)**  **1.99 (1.20-2.79)** | 1.48 (0.85-2.12)  1.40 (0.76-2.04)  1.61 (0.95-2.27)  **1.86 (1.12-2.61)** | 1.25 (0.79-1.71)  **1.65 (1.07-2.22)**  **1.75 (1.17-2.33)**  **1.88 (1.30-2.46)** | 1.20 (0.77-1.64)  **1.59 (1.03-2.16)**  **1.61 (1.07-2.15)**  **1.76 (1.21-2.31)** |
| Emotional / Informational Social Support  High (ref group)  Low |  | **1.77 (1.35-2.19)** |  | **1.66 (1.33-1.99)** |
|  |  |  |  |  |
